# Evaluation of the effects of different remineralizing agents on the microhardness and mineral content of bleached enamel along different storage times

**DOI:** 10.1186/s12903-026-07749-1

**Published:** 2026-02-17

**Authors:** Bassant Ismail, Zainab Soliman, Farid El-Askary

**Affiliations:** 1 Faculty of Dentistry, Badr University in Cairo, Cairo Suez Road, University Road, Cairo, 11829 Egypt; 2 Faculty of Dentistry, Ain Shams University, Cairo, Egypt

**Keywords:** In-office bleaching, Enamel remineralization, Microhardness, Mineral content analysis

## Abstract

**Objectives:**

This study evaluated the effects of various remineralizing agents on Vickers microhardness (HV) and mineral weight percentages (wt%) of bleached enamel during 30 days of storage in artificial saliva.

**Materials and methods:**

For HV testing, a total of 154 human specimens were used, including 14 specimens that were assigned to two control groups: a positive (sound unbleached enamel, *n* = 7) and a negative control (bleached using a 32% hydrogen peroxide, *n* = 7). The remaining 140 specimens were bleached and randomly divided into 20 experimental groups (*n* = 7), based on two factors: remineralizing agents; four agents (potassium nitrate with fluoride, casein-phosphopeptide-amorphous calcium phosphate with fluoride, hydroxyapatite with fluoride, and PAMAM), and storage times; five times (24 h, 3 days, 7 days, 15 days, and 30 days). For mineral wt%, 110 human enamel specimens were used, including 10 specimens for positive (*n* = 5) and negative control (*n* = 5), and 100 bleached enamel specimens divided into 20 experimental groups (*n* = 5/group), based on two factors, same as for HV testing. Data were analyzed using two-way ANOVA followed by Tukey’s HSD post hoc tests (α = 0.05).

**Results:**

Remineralizing agents and storage time significantly affected HV of bleached enamel (*p* < 0.001). PAMAM showed the highest HV at 7, 15, and 30 days (*p* < 0.05). Calcium (Ca) wt% was unaffected by either remineralizing agents (*p* = 0.848) or storage time (*p* = 0.141). Phosphorus (P) wt% was significantly influenced by both factors (*p* < 0.001 and *p* = 0.023, respectively). Fluoride (F) wt% was significantly affected only by the remineralizing agents (*p* = 0.001).

**Conclusions:**

Bleaching significantly decreased enamel HV and F wt%, whereas remineralizing agents effectively restored both.

**Clinical relevance:**

The use of different remineralizing agents may help counteract the detrimental effects of bleaching on enamel.

## Introduction

The growing aesthetic awareness among patients seeking dental treatments has increased the demand for whiter teeth, which can be conservatively achieved through tooth bleaching [1]. The primary active agents in vital tooth bleaching are hydrogen peroxide or carbamide peroxide [2]. Upon application, these agents release free radicals that diffuse into the enamel and dentin, breaking down chromogenic organic compounds and thereby reducing tooth discoloration [3]. However, once all available pigments have been oxidized and saturation is reached, bleaching may result in enamel protein degradation and mineral loss, leading to adverse effects on dental hard tissues [4–6]. Reported effects include increased enamel porosity, surface roughness, erosion, alterations in mineral content, and reduced microhardness, particularly when high-concentration peroxides or prolonged application times are used [7–11].

To mitigate the adverse effects of bleaching on enamel and dentin, several studies have recommended the use of various remineralizing agents during or after the bleaching process. These agents can aid in repairing enamel defects and restoring mineral loss induced by bleaching [7, 10, 12–14]. Additionally, they help reduce stain reuptake by restoring enamel surface integrity, decreasing surface roughness, and occluding pores formed during bleaching, thereby contributing to improved post-bleaching color stability [11, 14–16].

Fluoride, the most commonly used classical agent for enamel remineralization, is frequently applied in conjunction with bleaching procedures. It promotes enamel remineralization and enhances resistance to acid attacks by facilitating the formation of fluorapatite [10]. Previous studies have demonstrated its ability to restore mineral content and recover the microhardness of bleached enamel [17, 18]. However, its action is typically restricted to the superficial enamel layer, which limits the organized development of hydroxyapatite crystals in areas of deeper enamel demineralization [19, 20]. 

Casein phosphopeptide (CPP) is a bioactive compound from milk protein casein with great binding capacity to enamel, and is considered a saliva biomimetic, but with superior stabilization of calcium and phosphate ions due to its high phosphoseryl content [21]. When combined with amorphous calcium phosphate (ACP), with or without fluoride, they can form CPP-ACP and/or CPP-ACFP nanocomplexes that diffuse into enamel lesions, providing a reservoir of bioavailable remineralizing ions to repair enamel defects caused by bleaching [9, 22]. CPP-ACP-containing agents demonstrated greater remineralization potential compared to other remineralizing agents when applied to bleached enamel [9, 11].

Hydroxyapatite-based remineralizing agents have attracted significant interest due to their chemical similarity to enamel and dentin mineral content [11]. They have a high affinity to the enamel surface and were found to be effective remineralizing agents by supplying calcium and phosphate ions to areas of demineralization [23]. Their efficacy in post-bleaching remineralization has been documented [22, 24]. Moreover, Attia et al. [11], found that remineralizing agents containing hydroxyapatite (HAP) or CPP-ACP were more effective than fluoride-only treatments in reducing enamel surface roughness following bleaching procedures.

Although great evidence exists regarding the efficacy of conventional remineralizing agents for enamel surface remineralization, they fall short in rebuilding its organized crystal structure [25]. Recently, focus has shifted toward regenerative biomineralization therapies, which aim to repair damaged dental tissues using materials that closely mimic the action of enamel and dentin proteins to form highly organized mineralized structures [25]. Poly(amido amine) dendrimer (PAMAM), a hyper-branched analog of non-collagenous proteins, acts as an organic nucleation template, mimicking enamel proteins, to support biomimetic crystal growth on demineralized enamel [26]. In vitro studies have shown promising results for PAMAM in remineralizing artificially induced early enamel caries lesions [27, 28].

Although many laboratory and clinical studies have explored the impact of different remineralizing agents after bleaching, key gaps remain, especially in comparing traditional versus regenerative approaches. Research is limited on how these agents, particularly newer ones like PAMAM, perform after bleaching. Assessing and comparing the effects of various remineralizing agents with different mechanisms of action on enamel properties such as microhardness and mineral content over time could offer valuable insights into their clinical effectiveness.

Therefore, the aim of this in vitro study was to evaluate the effects of a chemically activated in-office bleaching agent on enamel microhardness and mineral content, and to investigate the influence of post-bleaching application of various remineralizing agents on bleached enamel over different storage periods. The null hypothesis tested was that neither bleaching nor the application of the remineralizing agents would affect enamel microhardness or mineral content.

## Materials and methods

This study used one chemically activated bleaching agent and four different remineralizing agents. Materials, description, composition, manufacturers, and lot numbers are listed in Table 1.

**Table 1:** Materials used in the study, their descriptions, compositions, manufacturers, and Lot#

Material	Description	Composition	Manufacturer	Lot #
Power Whitening YF (HP)	In-office chemically activated bleaching agent.	40% hydrogen peroxide (HP) (upon mixing HP concentration reaches 32%), polyglycol, organic amines, and silicon dioxide. (pH = 8-9.7).	WHITEsmile GmbH, Germany.	20044, 22062
After whitening mousse (KNO_3_ + F)	Gel.	30% Xylitol, 4.2% potassium nitrate, and 1450 ppm fluoride.	WHITEsmile GmbH, Germany.	20056
MI Paste Plus (CPP-ACPF)	Topical tooth cream.	Casein-phosphopeptide-amorphous calcium phosphate with 0.2% sodium fluoride (900 ppm), pure water, glycerol, d-sorbitol, CMC-Na, propylene glycol, silicon dioxide, titanium dioxide, xylitol, phosphoric acid, flavoring, sodium saccharin, ethyl, propyl, and butyl p-hydroxybenzoate.	GC America Inc., USA.	328619
Remin Pro (HAP + F)	Water-based cream.	Hydroxyapatite (calcium and phosphate), ethanoliccolophony, fluoride (1450 ppm NaF) and xylitol.	VOCO-GmbH, Germany.	327472
Polyamidoamine dendrimer (PAMAM)	Colorless liquid.	PAMAM-succinamic acid dendrimer, 1,4-diaminobutane core, generation 4 solution. 10 wt% in water.	Sigma-Aldrich Chemie GmbH, Germany.	Aldrich- 635871

### Sample size calculation

For microhardness (HV) testing, an a priori power analysis (G*Power v3.1.9.7) was performed for a two-factor factorial design (4 remineralizing agents × 5 storage times), requiring 20 experimental groups. Using the means and standard deviations of bleached and non-bleached enamel from a previous study [9], and an effect size of 0.62, 95% power, and α = 0.05, a sample of *n* = 7 specimens per agent–time (140 specimens in total) was required to detect differences among groups.

For the mineral weight percentage (wt%) analysis, a separate power calculation was performed based on the means and standard deviations of calcium levels (%) in bleached enamel with and without remineralizing agent treatment in a previous study [29], (effect size = 1.16, 95% power, α = 0.05), which indicated *n* = 5 specimens per agent–time, requiring 100 specimens in total for the 20 experimental groups.

### Collection of enamel specimens

A total of 94 freshly extracted sound human posterior teeth were retrieved from anonymous patients during routine dental procedures performed in the Oral and Maxillofacial Surgery Department in the “Sixth of October Central Hospital”, Giza, Egypt. The use of extracted human teeth in this study was approved by the Research Ethics Committee and Institutional Review Board of the Faculty of Dentistry, Ain Shams University (FDASU-REC), in May 2020, with approval number FDASU-RecID032004. The study adhered to the principles outlined in the Declaration of Helsinki. FDASU-REC approved the use of anonymized teeth and waived the requirement for individual patient consent, in accordance with national regulations.

Out of the 94 teeth, 55 were used for HV testing (including 33 premolars and 22 molars), while for mineral content testing, 39 teeth were used (including 22 premolars and 17 molars). After extraction, teeth were thoroughly cleaned under running water, and hard deposits were removed using an ultrasonic scaler (Woodpecker^®^ UDS-K LED, WOODPECKER, China).

Teeth crowns were cut at the cemento-enamel junction (CEJ) using slow-speed double-sided cutting abrasive discs (NTI^®^, Kerr dental, USA) mounted on a straight handpiece (NSK EX-6B, NSK, Japan) with a maximum speed of 40,000 rpm under copious water irrigation. Premolars’ crowns were divided into two halves, buccal and lingual, while molars’ crowns were sectioned into four quadrants, mesiobuccal, distobuccal, mesiolingual, and distolingual. Each enamel section served as an individual enamel specimen.

### Specimens preparation and grouping for HV testing

**Fig. 1:**
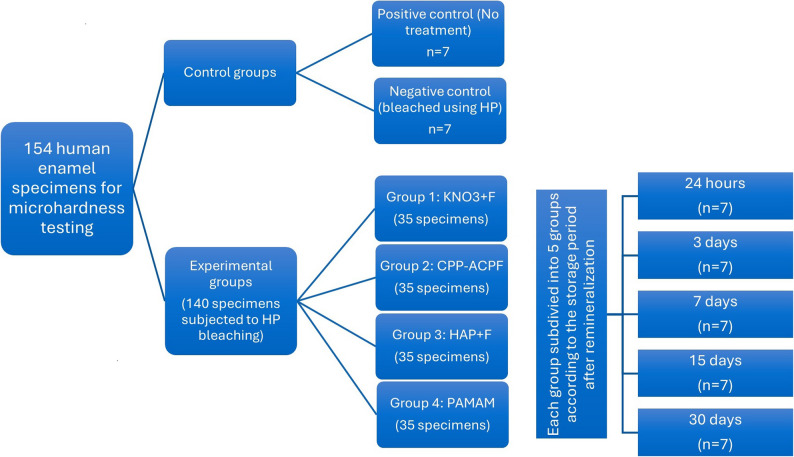
Study design and specimens’ distribution for HV testing

The 55 sound posterior teeth (33 premolars and 22 molars) assigned for HV testing were used to obtain a total of 154 enamel specimens. Fourteen specimens were used as two control groups positive control group, sound enamel without bleaching or remineralization, *n* = 7, and a negative control group, which was bleached without remineralization, *n* = 7. The remaining 140 specimens were randomly assigned to 20 experimental groups (*n* = 7 per group), based on two experimental factors: Level 1: four remineralizing agents (KNO3 + F, CPP-ACPF, HAP + F, and PAMAM) and Level 2: five storage periods (24 h, 3 days, 7 days, 15 days, and 30 days). Each group comprised 3 randomly selected premolar enamel specimens and 4 randomly selected molar enamel specimens (Fig. 1). The buccal and palatal/lingual surfaces of enamel specimens were wet ground using #600, #1000, #1500, and #2000 silicon carbide papers in a circular motion for 60 s each to create flat enamel surfaces prior to any treatment [30].

### Preparation of artificial saliva

Artificial saliva (AS) was prepared by dissolving 0.4 g NaCl, 0.4 g KCl, 0.795 g CaCl.2H_2_O, 0.78 g NaH_2_PO_4_.2H_2_O, 0.005 g Na_2_S.9H_2_O, and 1 g Urea in 1000 ml of warm distilled water. Potassium hydroxide (KOH) was finally added to neutralize the pH of the prepared saliva to reach a pH of 7 [31]. The AS solution was prepared in the laboratory of Analytical Chemistry at the Faculty of Pharmacy, Ain-Shams University.

### Bleaching procedure

The bleaching gel was dispensed from the barrel syringe using the auto-mixing tip attached to the dual syringe. The gel was applied directly to the surfaces of the prepared enamel specimens at a thickness of approximately 1–2 mm, following the manufacturer’s instructions. The gel was left undisturbed for 15 min, then removed with clean cotton. This procedure was repeated twice more with fresh gel each time, resulting in a total of three applications per specimen and a cumulative application time of 45 min. After completing the bleaching process, the specimens were thoroughly rinsed with distilled water to remove any residual gel.

### Application of the remineralizing agents

Immediately following the bleaching procedure, the specimens were blotted dry, and the application of the remineralizing agents was implemented according to the experimental group as follows:

The manufacturer’s recommended agent, containing KNO3 + F, was applied to the surface of enamel specimens and left for 10 min. To mimic the oral cavity, the specimens were placed in a tightly sealed container at 100% relative humidity and were kept at 37 °C inside an incubator (BT 1010, Biotech Company for Medical and Laboratory Equipment, Egypt) for 10 min, then the specimens were washed with distilled water.

The CPP-ACPF-containing remineralizing agent was applied to bleached enamel specimens according to the manufacturer’s instructions. A proper amount of the paste was distributed over the enamel by fingertips and left for 3 min at 100% relative humidity at 37 °C in a tightly sealed container inside the incubator. After 3 min, specimens were wetted with AS using a dental micro-brush and were left for 2 min in the same storage conditions.

to simulate the clinical situation. Specimens were wiped using clean cotton and were put in a tightly sealed container at 100% relative humidity/37°C for 30 min inside the incubator.

The HAP + F- containing remineralizing agent was applied by fingertips and was left for 3 min at 100% relative humidity at 37 °C. Specimens were wiped clean using a clean cotton and were placed in a tightly sealed container at 100% relative humidity for 30 min at 37 °C inside the incubator.

The PAMAM solution was applied to bleached enamel specimens using a dental micro-brush. The specimens were kept in a tightly sealed container at 100% relative humidity inside the incubator at 37 °C for 30 min based on previous studies [32, 33]. The specimens were monitored to allow reapplication of the solution if it dried on the specimen surface. Accordingly, PAMAM was reapplied once or twice on average, depending on need. After 30 min, the specimens were thoroughly rinsed with distilled water to remove any residual PAMAM solution. After finishing the remineralization procedures, the specimens were kept in artificial saliva (AS) inside the incubator set at 37 °C for the desired storage period. The AS was changed daily with a fresh solution.

Specimens in the positive control group (sound enamel) did not receive either bleaching or remineralization treatment, whereas specimens in the negative control group (bleached enamel) underwent bleaching treatment only. All specimens were stored in artificial saliva at 37 °C in an incubator for 24 h.

After each storage period, the specimens were washed with distilled water and air-dried using water-free compressed air before microhardness or mineral content testing.

### Vickers hardness (HV) test

After each storage period, specimens were fixed onto self-cured acrylic resin molds with the flattened enamel surface facing upwards, using cyanoacrylate adhesive. Vickers hardness (HV) was measured using a digital Vickers hardness tester (NEXUS4000™, INNOVATEST, model no. 4503, Netherlands) under a 100-gram load applied for 30 s [24]. The depth, area, and width of the symmetrically shaped indentations were measured using computer software under a microscope at 40× magnification. Three measurements were taken from each specimen and averaged. The mean HV (KgF) for each group (*n* = 7 specimens) was calculated and used for statistical analysis.

### Specimens’ grouping and preparation for mineral analysis

**Fig. 2:**
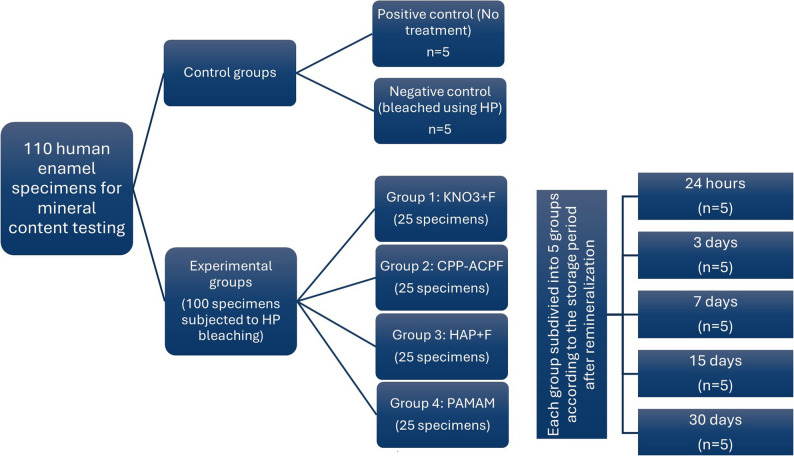
Study design and specimens’ distribution for mineral testing

The 39 extracted teeth (22 premolars and 17 molars) assigned for mineral analysis yielded 44 specimens from premolars and 68 specimens from molars. Since only 66 molar specimens were needed, the extra 2 molar specimens were discarded to obtain 110 enamel specimens. The specimens were polished using a non-fluoridated polishing paste (i-FASTE Prophy Paste, Douromed, EU) to remove surface debris and stains. Subsequently, all specimens were thoroughly rinsed with distilled water before treatment. Of the 110 specimens, 10 specimens were allocated to two control groups (positive control, *n* = 5, and negative control, *n* = 5). The remaining 100 specimens were divided into 20 experimental groups, based on two levels: the remineralizing agents (5 agents) and storage time (5 times), similar to the HV grouping but with 5 specimens per group (Fig. 2). The specimens were subjected to similar treatments to those of HV testing according to the assigned group.

### Evaluation of mineral weight percentages (wt%)

The mineral content was evaluated using a Scanning Electron Microscope attached to an energy-dispersive X-ray analyzer (SEM-EDX, Quanta FEG 250, FEI Company, Netherlands). Each specimen was analyzed under 150x magnification for the elemental distribution to measure the atomic and weight percentages of the Ca, P, and F elements. Three readings were taken of each specimen in 3 different locations. Each specimen served as a statistical unit for statistical analysis, and the average of the three readings/each specimen was calculated. The mean of the five specimens was calculated and used for statistical analyses.

### Statistical analysis

Data from both Vickers hardness and mineral content analyses were statistically processed using SPSS software for Windows (version 23, IBM Corporation, New York, USA). Normality of the data was assessed using the Shapiro-Wilk test, and homogeneity of variances was evaluated with Levene’s test. Comparisons between positive and negative control groups were performed using the Student’s t-test. One-way analysis of variance (ANOVA) followed by Dunnett’s test (two-sided) was employed to assess significant differences between each experimental group and the control groups. Two-way ANOVA with Tukey’s HSD post hoc test was used for pairwise comparisons among the experimental groups. Statistical significance was set at α = 0.05.

## Results

### Microhardness

Student t-test revealed that the Vickers hardness number (HVN) of bleached enamel was statistically significantly lower compared to that of sound enamel (Table 2, *p* = 0.025).

**Table 2:** Means ± SD of HVN of control groups and experimental groups

Positive control (Sound enamel)			Negative control (Bleached enamel)		
443.9 ± 54.5^Aa^			389.7 ± 13.1^Bµ^		
	KNO3 + F	CPP-ACPF		HAP + F	PAMAM
24 h	560.3 ± 36.7^b†^	480.2 ± 38.1^a†^		456.4 ± 104.9^aµ^	503.2 ± 58.5^b†^
3 days	508.6 ± 12.7^b†^	520.7 ± 9.8^b†^		412.2 ± 53.2^aµ^	510.9 ± 43.0^b†^
7 days	496.9 ± 13.5^b†^	512.8 ± 15.0^b†^		516.5 ± 5.0^a†^	545.7 ± 9.3^b†^
15 days	407.4 ± 58.2^aµ^	510.3 ± 40.1^b†^		529.3 ± 3.3^b†^	587.5 ± 4.1^b†^
30 days	578.2 ± 13.1^b†^	514.6 ± 6.8^b†^		419.6 ± 52.1^aµ^	651.4 ± 13.6^b†^

Means with different capital superscript letters indicate a statistically significant difference between positive & negative control groups at *p* = 0.05. Means with different small superscript letters and different symbols indicate statistically significant differences between experimental groups compared to positive and negative controls, respectively at *p* = 0.05

When comparing the experimental groups to the control groups, Table 2, the two-sided Dunnett’s test revealed that, for the KNO3 + F group, all storage times exhibited significantly higher HVN compared to both sound and bleached enamel (*p* < 0.05), except for the 15-day group, which showed no significant difference relative to either control (*p* > 0.05). CPP-ACPF demonstrated significantly higher HVN than sound enamel at all storage periods from 3 days onward (*p* < 0.05), and all HVN values were significantly greater than those of bleached enamel (*p* < 0.05). For the HAP + F group, only the 15-day group showed significantly higher HVN compared to sound enamel (*p* < 0.05), whereas the 7-day and 15-day groups exhibited significantly higher HVN than bleached enamel (*p* < 0.05). PAMAM consistently showed significantly higher HVN than both sound and bleached enamel at all storage times (*p* < 0.05).

Two-way ANOVA followed by Tukey’s HSD post hoc test revealed that “remineralizing agent”, “storage time”, and “remineralizing agent x storage time” showed a significant effect on the HVN of bleached enamel (*p* < 0.001).

Table 3 shows that, regarding the effects of the remineralizing agents, KNO3 + F exhibited significantly higher HVN than HAP + F at the 24-hour, 3-day, and 30-day storage intervals (*p* < 0.05). However, at 7 days and 15 days, it demonstrated significantly lower HVN than HAP + F and PAMAM (*p* < 0.05). CPP-ACPF showed significantly higher HVN compared to HAP + F at both 3-day and 30-day intervals (*p* < 0.05). Additionally, it exhibited significantly higher HVN than KNO3 + F at 15 days, whereas the reverse was observed at 30 days, with KNO3 + F group showing higher HVN (*p* < 0.05). HAP + F displayed the lowest HVN among all remineralizing agents at both the 3-day and 30-day storage times (*p* < 0.05). From 7 days onward, PAMAM consistently demonstrated the highest HVN among all agents tested (*p* < 0.05). At 24 h and 3 days, PAMAM did not show a significant difference compared to the other agents (*p* > 0.05), except in comparison to HAP + F at 3 days, where PAMAM exhibited significantly higher HVN (*p* < 0.05).

**Table 3:** Means ± SD of HVN of bleached enamel after application of the four remineralizing agents at each storage time

	KNO3 + F	CPP-ACPF	HAP + F	PAMAM
24 h	543.0 ± 50.5^Bc^	480.2 ± 22.2^ABa^	456.4 ± 104.9^Aab^	503.2 ± 58.5^ABa^
3 days	501.6 ± 24.0^Bb^	520.7 ± 9.8^Ba^	412.2 ± 53.2^Aa^	510.9 ± 43.0^Ba^
7 days	481.7 ± 27.4^Ab^	512.8 ± 15.0^ABa^	516.5 ± 5.0^Bb^	545.7 ± 9.3^Cab^
15 days	407.4 ± 58.2^Aa^	510.3 ± 40.1^Ba^	529.3 ± 3.3^Bb^	587.5 ± 4.1^Cb^
30 days	578.2 ± 13.1^Cc^	514.6 ± 6.8^Ba^	419.6 ± 52.1^Aa^	651.4 ± 13.6^Dc^

Within each row, means with different capital superscript letters are statistically significantly different at *p* = 0.05. Within each column, means with different small superscript letters are statistically significantly different at *p* = 0.05

Regarding the effect of storage duration (Table 3), KNO3 + F showed the highest HVN at the 30-day storage period, which was significantly greater than all other storage times except the 24-hour storage time (*p* < 0.05), while the 15-day storage period exhibited the lowest HVN (*p* < 0.05). For CPP-ACPF, no statistically significant differences in HVN were observed among the different storage times (*p* > 0.05). In the case of HAP + F, the 7-day and 15-day storage periods resulted in significantly higher HVN values compared to the 3-day and 30-day periods (*p* < 0.05). For PAMAM, the 30-day storage time produced the highest HVN, which was significantly greater than all other time points (*p* < 0.05).

### Mineral weight percentages

There was no statistically significant difference between the Ca wt% of sound and bleached enamel, or between the P wt% of sound and bleached enamel (Tables 4 and 5, *p* = 0.884). However, the F wt% was significantly higher in sound enamel compared to bleached enamel (Table 6, *p* = 0.015).

**Table 4:** Means ± SD of calcium (Ca) wt.% in the control and experimental groups

Positive control (Sound enamel)			Negative control (Bleached enamel)		
40.3 ± 2.3^Aa^			42.0 ± 3.2^Aµ^		
	KNO3 + F	CPP-ACPF		HAP + F	PAMAM
24 h	41.3 ± 6.1^aµ^	44.3 ± 4.9^aµ^		40.9 ± 7.7^aµ^	40.9 ± 3.9^aµ^
3 days	41.1 ± 5.2^aµ^	36.4 ± 6.0^aµ^		39.7 ± 3.6^aµ^	38.6 ± 5.8^aµ^
7 days	35.8 ± 3.8^aµ^	44.5 ± 7.0^aµ^		38.6 ± 8.0^aµ^	46.0 ± 4.6^aµ^
15 days	40.6 ± 9.2^aµ^	41.8 ± 6.0^aµ^		38.7 ± 4.6^aµ^	42.9 ± 2.6^aµ^
30 days	41.1 ± 3.9^aµ^	35.9 ± 4.3^aµ^		38.7 ± 2.1^aµ^	37.2 ± 3.9^aµ^

Means with different capital superscript letters indicate a statistically significant difference between positive & negative control groups at *p* = 0.05. Means with different small superscript letters and different symbols indicate statistically significant differences between experimental groups compared to positive and negative controls, respectively at *p* = 0.05

**Table 5:** Means ± SD of phosphorus (P) wt.% in the control groups and experimental groups

Positive control (Sound enamel)			Negative control (Bleached enamel)		
19.0 ± 1.0^Aa^			19.1 ± 1.2 ^Aµ^		
	KNO3 + F	CPP-ACPF		HAP + F	PAMAM
24 h	18.2 ± 1.4^aµ^	19.6 ± 1.4^aµ^		18.1 ± 3.3^aµ^	18.7 ± 0.9^aµ^
3 days	17.0 ± 1.8^aµ^	16.4 ± 0.4^b†^		16.7 ± 2.1^aµ^	18.7 ± 0.9^aµ^
7 days	17.1 ± 0.9^aµ^	18.3 ± 0.7^aµ^		14.6 ± 2.1^b†^	21.3 ± 2.0^b†^
15 days	15.4 ± 2.7^b†^	17.2 ± 1.0^aµ^		20.5 ± 1.0^aµ^	21.5 ± 1.0^b†^
30 days	18.7 ± 0.4^aµ^	17.2 ± 2.2^aµ^		18.2 ± 1.5^aµ^	18.3 ± 0.8^aµ^

Means with different capital superscript letters indicate a statistically significant difference between positive & negative control groups at *p* = 0.05. Means with different small superscript letters and different symbols indicate statistically significant differences between experimental groups compared to positive and negative controls, respectively at *p* = 0.05

**Table 6:** Means ± SD of fluorine (F) wt.% in the control groups and experimental groups

Positive control (Sound enamel)			Negative control (Bleached enamel)		
0.7 ± 0.3^Aa^			0.2 ± 0.2^Bµ^		
	KNO3 + F	CPP-ACPF		HAP + F	PAMAM
24 h	0.3 ± 0.2^aµ^	0.4 ± 0.3^aµ^		0.9 ± 0.5^a†^	0.3 ± 0.3^aµ^
3 days	0.9 ± 0.3^a†^	0.9 ± 0.2^a†^		1.2 ± 0.2^a†^	0.1 ± 0.1^bµ^
7 days	0.6 ± 0.3^aµ^	0.5 ± 0.2 ^aµ^		0.9 ± 0.2^a†^	0.5 ± 0.3^aµ^
15 days	1.4 ± 0.9^a†^	0.6 ± 0.1^a†^		0.3 ± 0.4^aµ^	0.7 ± 0.3^a†^
30 days	0.3 ± 0.1^aµ^	1.4 ± 0.1^b†^		0.7 ± 0.5^aµ^	0.7 ± 0.4^aµ^

Means with different capital superscript letters indicate a statistically significant difference between positive & negative control groups at *p* = 0.05. Means with different small superscript letters and different symbols indicate statistically significant differences between experimental groups compared to positive and negative controls, respectively at *p* = 0.05

Table 4 shows no significant differences in Ca wt% among the remineralizing agent groups, sound enamel, and bleached enamel at any storage time (*p* > 0.05). For P wt%, Table 5 shows that KNO3 + F at 15 days, CPP-ACPF at 3 days, and HAP + F at 7 days showed significantly lower values than sound and bleached enamel (*p* < 0.05). In contrast, PAMAM at 7 and 15 days exhibited significantly higher P wt% than both sound and bleached enamel (*p* < 0.05). Regarding F wt%, Table 6 shows that KNO3 + F at 3 and 15 days demonstrated significantly higher values than bleached enamel (*p* < 0.05). CPP-ACPF at 30 days showed significantly higher F wt% than sound enamel (*p* < 0.05), while at 3, 7, and 30 days, it showed significantly higher F wt% than bleached enamel (*p* < 0.05). HAP + F at 24 h, 3, and 7 days had significantly higher F wt% than bleached enamel (*p* < 0.05). PAMAM at 3 days had significantly lower F wt% than sound enamel, while at 15 days it was significantly higher than that of bleached enamel (*p* < 0.05).

Regarding the effect of remineralizing agents on bleached enamel, two-way ANOVA showed that Ca wt% was not significantly affected by the remineralizing agents (*p* = 0.848), storage time (*p* = 0.141), or their interaction (*p* = 0.106). P wt% was significantly affected by the remineralizing agents (*p* < 0.001), storage times (*p* = 0.023), and their interaction (*p* < 0.001). F wt% was significantly affected by the remineralizing agents (*p* = 0.001) and the interaction between remineralizing agents and storage time (*p* < 0.001).

Table 7 shows that, regarding the effects of remineralizing agents on the Ca wt.% of bleached enamel, no significant differences between the four remineralizing agents were found (*p* > 0.05). As regards the effect of storage time, PAMAM showed a significantly higher Ca wt% at 7 days compared to 30 days (*p* < 0.05).

**Table 7:** Means ± SD of Ca, P, and F wt% of the experimental groups at each storage time

		KNO3 + F	CPP-ACPF	HAP + F	PAMAM
Calcium	24 h	41.3 ± 6.1^Aa^	44.3 ± 4.9^Aa^	40.9 ± 7.7^Aa^	40.9 ± 3.9^Aab^
	3 days	41.1 ± 5.2^Aa^	36.4 ± 6.0^Aa^	39.7 ± 3.6^Aa^	38.6 ± 5.8^Aab^
	7 days	35.8 ± 3.8^Aa^	44.5 ± 7.0^Aa^	38.6 ± 8.0^Aa^	46.0 ± 4.6^Ab^
	15 days	40.6 ± 9.2^Aa^	41.8 ± 6.0^Aa^	38.7 ± 4.6^Aa^	42.9 ± 2.6^Aab^
	30 days	41.1 ± 3.9^Aa^	35.9 ± 4.3^Aa^	38.7 ± 2.1^Aa^	37.2 ± 3.9^Aa^
Phosphorus	24 h	18.2 ± 1.4^Aab^	19.6 ± 1.4^Ab^	18.1 ± 3.3^Aab^	18.7 ± 0.9^Aa^
	3 days	17.0 ± 1.8^Aab^	16.4 ± 0.4^Aa^	16.7 ± 2.1^Aab^	18.7 ± 0.9^Aa^
	7 days	17.1 ± 0.9^ABab^	18.3 ± 0.7^Bab^	14.6 ± 2.1^Aa^	21.3 ± 2.0^Cb^
	15 days	15.4 ± 2.7^Aa^	17.2 ± 1.0^Aa^	20.5 ± 1.0^Bb^	21.5 ± 1.0^Bb^
	30 days	18.7 ± 0.4^Ab^	17.2 ± 2.2^Aab^	18.2 ± 1.5^Aab^	18.3 ± 0.8^Aa^
Fluorine	24 h	0.3 ± 0.2^Aa^	0.4 ± 0.3^Aa^	0.9 ± 0.5^Aab^	0.3 ± 0.3^Aab^
	3 days	0.9 ± 0.3^Bab^	0.9 ± 0.2^Bb^	1.2 ± 0.2^Bb^	0.1 ± 0.1^Aa^
	7 days	0.6 ± 0.3^Aab^	0.5 ± 0.2^Aa^	0.9 ± 0.2^Aab^	0.5 ± 0.3^Aab^
	15 days	1.4 ± 0.9^Bb^	0.6 ± 0.1^ABab^	0.3 ± 0.4^Aa^	0.7 ± 0.3^ABb^
	30 days	0.3 ± 0.1^Aa^	1.4 ± 0.1^Bc^	0.7 ± 0.5^Aab^	0.7 ± 0.4^Ab^

Within each row, means with different capital superscript letters are statistically significantly different at *p* = 0.05. Within each column, for each element, means with different small superscript letters are statistically significantly different at *p* = 0.05

Regarding the effect of remineralizing agents on the P wt% of bleached enamel (Table 7), KNO3 + F and CPP-ACPF showed significantly lower values than PAMAM at both 7 and 15 days (*p* < 0.05). HAP + F exhibited significantly lower values than both CPP-ACPF and PAMAM at 7 days (*p* < 0.05), but at 15 days, it had significantly higher values than KNO3 + F and CPP-ACPF (*p* < 0.05). PAMAM demonstrated the highest P wt% among all agents at 7 days (*p* < 0.05), and significantly exceeded KNO3 + F and CPP-ACPF at 15 days (*p* < 0.05). Concerning storage time (Table 7), KNO3 + F at 30 days showed significantly higher P wt% than at 15 days (*p* < 0.05). CPP-ACPF at 24 h had significantly higher values than at 3 and 15 days (*p* < 0.05). HAP + F exhibited significantly higher values at 15 days compared to 7 days (*p* < 0.05). PAMAM showed the highest values at 7 and 15 days compared to other time points (*p* < 0.05).

Regarding F wt% (Table 7), KNO3 + F showed significantly higher values than PAMAM at 3 days and HAP + F at 15 days (*p* < 0.05), but significantly lower values than CPP-ACPF at 30 days (*p* < 0.05). CPP-ACPF recorded significantly higher values than PAMAM only at 3 days and exhibited the highest F wt% among all agents at 30 days (*p* < 0.05). HAP + F had a higher F wt% than PAMAM at 3 days but lower values than KNO3 + F at 15 days and CPP-ACPF at 30 days (*p* < 0.05). PAMAM demonstrated the lowest F wt% compared to all other agents at 3 days and remained significantly lower than CPP-ACPF at 30 days (*p* < 0.05). With respect to storage time (Table 3), KNO3 + F showed significantly higher values at 15 days compared to 24 h and 30 days (*p* < 0.05). CPP-ACPF reached the highest F wt% at 30 days compared to earlier time points (*p* < 0.05). HAP + F exhibited a significantly higher value at 3 days than at 15 days (*p* < 0.05). PAMAM showed significantly higher F wt% at 15 and 30 days compared to 3 days (*p* < 0.05).

## Discussion

Although dental bleaching improves tooth esthetics, its adverse effects are well-documented [22]. In-office bleaching, in particular, can alter enamel hardness and microstructure due to the high concentration of peroxide and the acidic pH of many commercial bleaching agents [34]. These alterations may compromise clinical outcomes by increasing surface roughness and susceptibility to staining [35]. The use of a 32% H₂O₂ in-office bleaching gel in this study was based on its proven efficacy in producing rapid and noticeable shade changes, making it a preferred option over home bleaching products [36, 37]. To replicate clinical conditions, remineralizing agents were applied immediately after bleaching. All enamel specimens, whether sound, bleached, or treated with remineralizing agents, were stored in artificial saliva, which was refreshed daily to simulate the oral environment [17].

Vickers microhardness (HV) testing is widely regarded as a suitable and practical method for detecting and quantifying surface enamel changes resulting from bleaching and the subsequent application of remineralizing agents [24, 38, 39]. It has been employed in numerous studies to indirectly evaluate the degree of enamel mineralization [9, 17, 40]. Energy Dispersive X-ray Analysis (EDX) is another technique used to assess changes in mineral content, specifically elements such as calcium, phosphorus, and fluorine, that influence the mechanical properties of enamel [41, 42]. EDX is valued for its rapid analysis, sensitivity to trace elements, and ability to map elemental distributions at the microscopic level, making it highly applicable in dental research [43].

In the present study, the null hypothesis stating that bleaching has no effect on enamel microhardness was rejected. However, the hypothesis concerning its effect on mineral content was partially accepted, as only the F wt% was significantly affected. The results demonstrated that bleaching led to a significant reduction in enamel microhardness, which aligns with previous findings [9, 12, 17, 39]. It was previously reported that the reduction in enamel HV may be attributed to mineral loss induced by the redox activity of hydrogen peroxide, which interacts with the enamel’s organic matrix, particularly at high concentrations or with repeated applications, resulting in mineral dissolution [4, 44]. However, this explanation was not fully supported by the findings of the present study, as no significant changes were observed in the wt% of Ca or P. In contrast, a significant decrease in F wt% was detected, suggesting that fluoride loss may be a critical factor influencing enamel hardness. Fluoride is known to enhance enamel stability by promoting the formation of fluorapatite, which possesses greater microhardness than hydroxyapatite [45].

The application of remineralizing agents resulted in the recovery of enamel microhardness to levels comparable to, or even exceeding, those of sound enamel at all storage durations. However, it induced significant changes in the wt% of P and F, with no significant changes in the Ca wt% of bleached enamel. Consequently, the null hypothesis stating that “remineralizing agents do not affect enamel microhardness” was rejected. However, the hypothesis regarding the absence of an effect on the mineral content of bleached enamel was partially accepted.

The recovery of bleached enamel microhardness may be attributed to the effectiveness of remineralizing agents in repairing bleaching-induced defects through classical, biomimetic, or combined remineralization mechanisms. In the first remineralization group, the manufacturer’s recommended remineralizing agent was used, which contains sodium fluoride, which, at high concentrations, forms a calcium fluoride-rich layer on the enamel surface, facilitating subsequent fluoride diffusion into subsurface enamel defects [10]. A previous study has demonstrated fluoride’s ability to enhance enamel microhardness post-bleaching to levels exceeding those of unbleached sound enamel [18]. This improvement may result from the partial substitution of hydroxyapatite (HAP) crystals with fluorapatite (FAP), which has been shown to exhibit greater microhardness than HAP [45].

The remineralizing effect of CPP-ACP–based remineralizing agents, such as the agent used in the second group, MI Paste Plus, is attributed to the ability of casein phosphopeptides to stabilize calcium and phosphate ions. The amorphous calcium phosphate (ACP) nanocomplexes, which act as Ca and P reservoirs, adhere to tooth surfaces, promoting mineral deposition [9]. Additionally, the presence of sodium fluoride in this agent enhances its remineralizing efficacy through a synergistic effect, forming ACPF complexes, further improving its post-bleaching remineralization potential [11, 46].

In the third group, Remin Pro, which contains nano-hydroxyapatite (nano-HAP) and sodium fluoride (HAP + F), was used. The nano-HAP closely mimics the structure, morphology, and crystallinity of enamel HAP and, due to its small particle size, can function as a filler to repair surface defects [25]. Previous studies have demonstrated the effectiveness of HAP + F in enhancing microhardness and reducing surface damage in bleached enamel [22, 24].

On the other hand, in the fourth group, PAMAM was used, which is a biomimetic remineralizing agent that can self-assemble in aqueous solution into macroscopic aggregates that mimic the behavior of natural amelogenin [47]. The positively charged PAMAM dendrimers bind to the negatively charged enamel surface and attract calcium and phosphate ions, facilitating the formation of new HAP nanorods with structural, orientational, and mineral phase characteristics similar to those of sound enamel [47]. Although no prior studies have specifically evaluated its effect on bleached enamel, Deokar et al. reported that PAMAM was more effective than sodium fluoride in restoring enamel microhardness in initial carious lesions [28]. Similarly, Khater et al. found that PAMAM achieved greater microhardness recovery in demineralized enamel after four weeks compared to nano-HAP [27].

Regarding the effect of each remineralizing agent over the storage periods, KNO3 + F demonstrated the highest HVN at both 24 h and 30 days post-bleaching. These time points also showed the highest numerical values for Ca and P wt%, with a statistically significant increase in P wt% observed at 30 days. The rapid recovery of HVN at 24 h may be attributed to the high affinity of freshly bleached enamel for remineralizing ions, which facilitates early microhardness recovery [13]. However, the subsequent decline in HVN from day 3 to day 15 could be explained by the transient nature of fluoride ions binding to enamel, particularly given the short contact time of KNO3 + F following bleaching [13]. Additionally, residual hydrogen peroxide, which can persist in enamel and dentin for up to two weeks post-bleaching [13], may have interfered with the remineralization process. By 30 days, the residual hydrogen peroxide had likely dissipated, allowing for more effective mineral uptake from artificial saliva and promoting further recovery of enamel microhardness.

On the other hand, CPP-ACPF was able to maintain stable microhardness values throughout the entire storage period, consistent with findings from previous studies [9, 22, 40, 48]. This effect can be attributed to the ability of CPP to stabilize calcium and phosphate ions on the bleached enamel surface, thereby serving as a sustained reservoir of remineralizing minerals [48]. Poggio et al. [22] reported that bleached enamel treated with CPP-ACPF exhibited a homogeneous, thick, and uniform layer of HAP, with less evidence of bleaching-induced damage upon scanning electron microscopy examination.

HAP + F showed the highest HVN values at 7 and 15 days post-bleaching, followed by a decrease at 30 days to values that were statistically similar to those at 24 h and 3 days post-bleaching. This fluctuation in HVN may be explained by the instability of the newly deposited HAP crystals, with the subsequent dissolution of minerals, which could allow mineral precipitation from artificial saliva, causing an increase in HVN. However, this could be followed by the loss of such minerals after reaching a certain saturation point [49]. It is also possible that the short application time of HAP + F did not initially allow proper penetration and stable binding of minerals to enamel defects, due to agglomeration of the nanosized HAP, suggesting that repeated or prolonged application could be more beneficial [13, 50]. Previous studies showed that HAP + F remineralizing agent resulted in areas not completely covered by the HAP layer when used after bleaching, demonstrating inferior remineralizing properties [22].

When evaluating the effect of PAMAM on enamel HV over the storage periods, values were observed to increase progressively, ultimately exceeding the typical range reported for sound enamel (270–360 HVN) and the mean value of 443.9 reported in the current study [24]. This enhancement may be attributed to PAMAM’s biomimetic remineralizing action, which mimics the function of natural enamel proteins [33]. Although PAMAM itself does not contain remineralizing minerals, storage of specimens in artificial saliva, rich in calcium and phosphorus ions, likely contributed to the gradual increase in HVN over time [27]. PAMAM acts as a scaffold that facilitates the nucleation and growth of HAP crystals from the surrounding medium, forming structures that closely resemble the size, orientation, and mineral phase of native enamel [27, 28, 32]. In addition, its electrostatic interaction with the negatively charged enamel surface enhances the targeted deposition of calcium and phosphate ions, thereby promoting effective remineralization [51]. PAMAM also improves the adhesion of the newly formed mineral layer to the enamel substrate, which may account for the significant increase in microhardness observed [33]. This finding is consistent with the study by Deokar et al., who reported a gradual increase in enamel microhardness in PAMAM-treated artificially demineralized enamel over time [27].

In addition to the distinct remineralization mechanisms of each agent, differences in application time and frequency, which can influence an agent’s contact time with enamel and thereby affect its binding to the enamel surface and overall remineralization efficacy [13], may also have contributed to the variation observed among the remineralization groups.

Despite variations in HVN, changes in mineral wt% were minimal, suggesting a weak correlation between mineral content and enamel microhardness. EDX is a semiquantitative technique with inherent limitations, including relatively large error margins that can reach ± 5%, which may be further exacerbated by surface roughness [52]. EDX provides relative elemental percentages rather than absolute concentrations and does not offer information on the chemical compounds or their structural arrangements, both of which can influence the physical properties of enamel. Enamel samples with similar mineral content may exhibit differing microhardness values due to variations in HAP crystal size, orientation, and organic matrix composition [41]. Furthermore, the detection depth of EDX is limited to approximately 2–5 microns, whereas the indentation depth from microhardness testing can exceed this range and is affected by the underlying enamel microstructure [53]. In addition, EDX specimens in the present study were polished without complete surface flattening, preserving the hypermineralized surface layer. In contrast, the specimens prepared for microhardness testing were ground to achieve a flat surface, which may have influenced the differential effects observed during bleaching and remineralization processes [53].

The current study has several limitations. The enamel specimens were stored under ideal remineralization conditions without exposure to pH fluctuations, which do not reflect the dynamic conditions of the oral environment [14, 54]. Incorporating pH cycling protocols could offer a more accurate assessment of the efficacy of remineralizing agents under conditions that better simulate intraoral challenges. Additionally, applying identical specimen preparation protocols for both HV and EDX evaluations could enhance the comparability of results and improve the correlation between microhardness and mineral content data. Moreover, the use of cross-sectional microhardness testing could provide more comprehensive insights into the depth-related effects of bleaching and subsequent remineralization treatments.

## Conclusions

Within the limitations of this study, the following could be concluded:

1. A 32% hydrogen peroxide bleaching agent negatively affected enamel microhardness and F wt%.
2. Calcium was the only mineral that was not significantly affected by either the bleaching procedure or the application of the various remineralizing agents.
3. The application of various remineralizing agents to bleached enamel restored enamel microhardness and partially recovered P and F wt%; however, these effects appeared to be dependent on both the type of remineralizing agent and the storage duration.

## Data Availability

The datasets used and/or analyzed during the current study are available from the corresponding author upon reasonable request.

## Abbreviations

HV: Vickers microhardness
wt%: Weight percentage
PAMAM: Polyamidoamine dendrimer
CPP-ACPF: Casein phosphopeptide-amorphous calcium phosphate with fluoride
KNO_3_: Potassium nitrate
HAP: Hydroxyapatite
AS: Artificial saliva
SEM: Scanning Electron Microscope
EDX: Energy-dispersive X-ray analysis
FAP: Fluorapatite

## References

[1] da Silva FB, Chisini LA, Demarco FF, et al. Desire for tooth bleaching and treatment performed in Brazilian adults: findings from a birth cohort. Braz Oral Res. 2018;32:1–10. 10.1590/1807-3107bor-2018.vol32.0012. 10.1590/1807-3107bor-2018.vol32.0012 29538477

[2] Fioresta R, Melo M, Forner L, Sanz JL. Prognosis in home dental bleaching: a systematic review. Clin Oral Investig. 2023;27:3347–61. 10.1007/s00784-023-05069-0. 37273018 10.1007/s00784-023-05069-0 PMC10329590

[3] Irusa K, Alrahaem IA, Ngoc CN, Donovan T. Tooth whitening procedures: a narrative review. Dent Rev. 2022;2:100055. 10.1016/j.dentre.2022.100055.

[4] Kwon SR, Wertz PW. Review of the mechanism of tooth whitening. J Esthet Restor Dent. 2015;27:240–57. 10.1111/jerd.12152. 25969131 10.1111/jerd.12152

[5] Ribeiro JS, Barboza AS, Cuevas-Suárez CE, et al. Novel in-office peroxide-free tooth-whitening gels: bleaching effectiveness, enamel surface alterations, and cell viability. Sci Rep. 2020;10:66733. 10.1038/s41598-020-66733-z. 10.1038/s41598-020-66733-z PMC7308351 32572064

[6] Patil G, Reche A, Paul P. Tooth bleaching and its adverse effects: a review. J Pharm Res Int. 2022;34:35–44. 10.9734/jpri/2022/v34i577257.

[7] Samaha A, Gomaa D. The effect of different remineralizing agents on laser bleached enamel. Egypt Dent J. 2020;66:469–83. 10.21608/edj.2020.79123.

[8] Krishnakumar K, Tandale A, Mehta V, et al. Post-operative sensitivity and color change due to in-office bleaching with the prior use of different desensitizing agents: a systematic review. Cureus. 2022;14:e24028. 10.7759/cureus.24028. 35547454 10.7759/cureus.24028 PMC9090214

[9] Melo M, Fioresta R, Sanz JL, et al. Effect of highly concentrated bleaching gels on enamel microhardness and superficial morphology, and the recovery action of four remineralizing agents. BMC Oral Health. 2022;22:693. 10.1186/s12903-022-02693-2. 10.1186/s12903-022-02693-2 PMC9793581 36575416

[10] Dionysopoulos D, Koliniotou-Koumpia E, Tolidis K, et al. Effect of fluoride treatments on bleached enamel microhardness and surface morphology. Oral Health Prev Dent. 2017;15:169–75. 10.3290/j.ohpd.a37929. 28322361 10.3290/j.ohpd.a37929

[11] Attia R, Kamel M. Changes in surface roughness of bleached enamel by using different remineralizing agents. Tanta Dent J. 2016;13:179–86. 10.4103/1687-8574.195707.

[12] Pizani AMA, Tholt B, Paciornik S, et al. Dental bleaching agents with calcium and their effects on enamel microhardness and morphology. Braz J Oral Sci. 2015;14:154–8. 10.1590/1677-3225v14n2a11.

[13] Da Costa Soares MUS, Araújo NC, Borges BCD, et al. Impact of remineralizing agents on enamel microhardness recovery after in-office tooth bleaching therapies. Acta Odontol Scand. 2013;71:343–8. 10.3109/00016357.2012.681119. 22564069 10.3109/00016357.2012.681119

[14] Bilge K, Kılıç V. Effects of different remineralizing agents on color stability and surface characteristics of the teeth following vital bleaching. Microsc Res Tech. 2021;84:2206–18. 10.1002/jemt.23774. 33852758 10.1002/jemt.23774

[15] Monteiro DDH, Valentim PT, Elias DC, et al. Effect of surface treatments on staining and roughness of bleached enamel. Indian J Dent Res. 2019;30:393–8. 10.4103/ijdr.IJDR_233_16. 31397414 10.4103/ijdr.IJDR_233_16

[16] Ertemür A, Yazkan B. Effect of different remineralization agents on the optical properties of bleached enamel. BMC Oral Health. 2025;25:542. 10.1186/s12903-025-05923-5. 40217272 10.1186/s12903-025-05923-5 PMC11992821

[17] Scribante A, Poggio C, Gallo S, et al. In vitro re-hardening of bleached enamel using mineralizing pastes: toward preventing bacterial colonization. Materials. 2020;13:818. 10.3390/ma13040818. 32054090 10.3390/ma13040818 PMC7079603

[18] Mousa E, Abdel-Fattah WM, Afifi R. Surface microhardness of bleached teeth enamel following different remineralizing approaches (in-vitro study). Alex Dent J. 2023;48:139–45. 10.21608/adjalexu.2022.130119.1266.

[19] Batra A, Shetty V. Non-fluoridated remineralizing agents: a review of literature. J Evol Med Dent Sci. 2021;10:638–44. 10.14260/jemds/2021/136.

[20] Arifa MK, Ephraim R, Rajamani T. Recent advances in dental hard tissue remineralization: a review of literature. Int J Clin Pediatr Dent. 2019;12:139–44. 10.5005/jp-journals-10005-1603. 31571787 10.5005/jp-journals-10005-1603 PMC6749882

[21] Cochrane NJ, Cai F, Huq NL, et al. Critical review in oral biology & medicine: new approaches to enhanced remineralization of tooth enamel. J Dent Res. 2010;89:1187–97. 10.1177/0022034510376046. 20739698 10.1177/0022034510376046

[22] Poggio C, Grasso N, Ceci M, et al. Ultrastructural evaluation of enamel surface morphology after tooth bleaching followed by the application of protective pastes. Scanning. 2016;38:221–6. 10.1002/sca.21263. 26376339 10.1002/sca.21263

[23] Meyer F, Enax J, Amaechi BT, et al. Hydroxyapatite as remineralization agent for children’s dental care. Front Dent Med. 2022;3:859560. 10.3389/fdmed.2022.859560.

[24] Kamath U, Sheth H, Mullur D, Soubhagya M. The effect of remin Pro^®^ on bleached enamel hardness: an in-vitro study. Indian J Dent Res. 2013;24:690–3. 10.4103/0970-9290.127612. 24552928 10.4103/0970-9290.127612

[25] Philip N. State of the Art enamel remineralization systems: the next frontier in caries management. Caries Res. 2019;53:284–95. 10.1159/000493031. 30296788 10.1159/000493031 PMC6518861

[26] Fan M, Zhang M, Xu HHK, et al. Remineralization effectiveness of the PAMAM dendrimer with different terminal groups on artificial initial enamel caries in vitro. Dent Mater. 2020;36:210–20. 10.1016/j.dental.2019.11.015. 31785833 10.1016/j.dental.2019.11.015

[27] Khater AA, Jamil WE, Gad NA. The effect of Poly Amido amine dendrimer, nano-hydroxyapatite and their combination on microhardness of demineralized enamel. Al-Azhar J Dent. 2023;10:94–100. 10.58675/2974-4164.1587.

[28] Deokar V, Mandale MS, Humbe JG, et al. A comparative assessment of remineralization potential of sodium fluoride (NaF) and Poly Amido amine (PAMAM) on artificial caries-like lesion of enamel: an in vitro study. Medicon Dent Sci. 2023;3:10–55162. 10.55162/mcds.03.058.

[29] Gjorgievska E, Nicholson JW. Prevention of enamel demineralization after tooth bleaching by bioactive glass incorporated into toothpaste. Aust Dent J. 2011;56:193–200. 10.1111/j.1834-7819.2011.01323.x. 21623812 10.1111/j.1834-7819.2011.01323.x

[30] Sa Y, Wang Z, Ma X, et al. Investigation of three home-applied bleaching agents on enamel structure and mechanical properties: an in situ study. J Biomed Opt. 2012;17:035002. 10.1117/1.jbo.17.3.035002. 22502559 10.1117/1.JBO.17.3.035002

[31] Pytko-Polonczyk J, Jakubik A, Przeklasa-Bierowiec A, Muszynska B. Artificial saliva and its use in biological experiments. J Physiol Pharmacol. 2017;68:807–13. 29550792

[32] Chen M, Yang J, Li J, et al. Modulated regeneration of acid-etched human tooth enamel by a functionalized dendrimer that is an analog of amelogenin. Acta Biomater. 2014;10:4437–46. 10.1016/j.actbio.2014.05.016. 24879313 10.1016/j.actbio.2014.05.016

[33] Li J, Chen L, Liang K, et al. Regeneration of biomimetic hydroxyapatite on etched human enamel by anionic PAMAM template in vitro. Arch Oral Biol. 2013;58:975–80. 10.1016/j.archoralbio.2013.03.008. 23598056 10.1016/j.archoralbio.2013.03.008

[34] Misilli T, Çarıkçıoğlu B, Deniz Y, Aktaş Ç. The impact of remineralization agents on dental bleaching efficacy and mineral loss in bleached enamel. Eur J Oral Sci. 2022;130:e12905. 10.1111/eos.12905. 36349560 10.1111/eos.12905

[35] Goyal K, Saha SG, Bhardwaj A, et al. A comparative evaluation of the effect of three different concentrations of in-office bleaching agents on microhardness and surface roughness of enamel: an in vitro study. Dent Res J. 2021;18:49. 10.4103/1735-3327.318944. PMC8351945 34429869

[36] Majeed A, Farooq I, Grobler SR, Rossouw RJ. Tooth-bleaching: a review of the efficacy and adverse effects of various tooth whitening products. J Coll Physicians Surg Pak. 2015;25:891–6. 26691365

[37] Kury M, Prunes BB, Saraceni CHC, et al. Clinical decision-making in tooth bleaching based on current evidence: a narrative review. Dent Mater. 2025;41:536–52. 10.1016/j.dental.2025.03.002. 40082147 10.1016/j.dental.2025.03.002

[38] Mushashe AM, Coelho BS, Garcia PP, et al. Effect of different bleaching protocols on whitening efficiency and enamel superficial microhardness. J Clin Exp Dent. 2018;10:e772–5. 10.4317/jced.54967. 30305875 10.4317/jced.54967 PMC6174019

[39] Mondelli RFL, Gabriel TRC, Rizzante FAP, et al. Do different bleaching protocols affect the enamel microhardness? Eur J Dent. 2015;9:25–30. 10.4103/1305-7456.149634. 25713480 10.4103/1305-7456.149634 PMC4319295

[40] Kaur G, Sanap AU, Aggarwal SD, Kumar T. Comparative evaluation of two different remineralizing agents on the microhardness of bleached enamel surface: results of an in vitro study. Indian J Dent Res. 2015;26:176–9. 10.4103/0970-9290.159154. 26096113 10.4103/0970-9290.159154

[41] Mansoor A, Moeen F, Mehmood M, et al. Micro-hardness and mineral content in the healthy tooth enamel. Pak Armed Forces Med J. 2019;69:1204–9.

[42] Poorni S, Kumar RA, Shankar P, et al. Effect of 10% sodium ascorbate on the calcium:phosphorus ratio of enamel bleached with 35% hydrogen peroxide: an in vitro quantitative energy-dispersive X-ray analysis. Contemp Clin Dent. 2010;1:223. 10.4103/0976-237x.76388. 22114425 10.4103/0976-237X.76388 PMC3220141

[43] Scimeca M, Bischetti S, Lamsira HK, et al. Energy dispersive X-ray (EDX) microanalysis: a powerful tool in biomedical research and diagnosis. Eur J Histochem. 2018;62:89–99. 10.4081/ejh.2018.2841. 10.4081/ejh.2018.2841 PMC5907194 29569878

[44] Rastelli AN, Nicolodelli G, Romano RA, et al. After bleaching enamel remineralization using a bioactive glass-ceramic (BioSilicate^®^). Biomed Glasses. 2016;2:1–7. 10.1515/bglass-2016-0001.

[45] Pajor K, Pajchel L, Kolmas J. Hydroxyapatite and fluorapatite in Conservative dentistry and oral implantology: a review. Materials. 2019;12:2683. 10.3390/ma12172683. 31443429 10.3390/ma12172683 PMC6747619

[46] Hamed N, Nasser M, Ghallab. The effect of different remineralizing agents on microleakage around restored demineralized enamel: an in vitro comparative study. Ain Shams Dent J. 2023;32:26–36. 10.21608/asdj.2023.180541.1160.

[47] Tao S, Fan M, Xu HHK, et al. The remineralization effectiveness of PAMAM dendrimer with different terminal groups on demineralized dentin in vitro. RSC Adv. 2017;7:54947–55. 10.1039/c7ra11844a.

[48] Valian A, Salehi EM, Samadi I, et al. Effects of CPP-ACP and Remin-Pro on surface roughness of bleached enamel: an atomic force microscopy study. J Dent Sch. 2018;38:74–8. 10.22037/jds.v38i2.32175.

[49] Enax J, Fandrich P, Schulze zur Wiesche E, Epple M. The remineralization of enamel from saliva: a chemical perspective. Dent J (Basel). 2024;12:339. 10.3390/dj12110339. 39590389 10.3390/dj12110339 PMC11592461

[50] Cai Z, Wang X, Zhang Z, et al. Large-scale and fast synthesis of nano-hydroxyapatite powder by a microwave-hydrothermal method. RSC Adv. 2019;9:13623–30. 10.1039/c9ra00091g. 35519585 10.1039/c9ra00091g PMC9063869

[51] Farooq I, Bugshan A. The role of salivary contents and modern technologies in the remineralization of dental enamel: a review. F1000Res. 2021;9:171. 10.12688/f1000research.22499.1. 10.12688/f1000research.22499.1 PMC7076334 32201577

[52] Carlton RA, Lyman CE, Roberts JE. Accuracy and precision of quantitative energy-dispersive X-ray spectrometry in the environmental scanning electron microscope. Scanning. 2004;26:167–74. 10.1002/sca.4950260404. 15473268 10.1002/sca.4950260404

[53] Fernando JR, Walker GD, Park TKS, et al. Comparison of calcium-based technologies to remineralise enamel subsurface lesions using microradiography and microhardness. Sci Rep. 2022;14:9888. 10.1038/s41598-022-13905-8. 10.1038/s41598-022-13905-8 PMC9197824 35701508

[54] Jablonski-Momeni A, Lentz J, Jablonski B, et al. A comparison between in vitro and randomized in situ models for remineralization of artificial enamel lesions. Sci Rep. 2024;14:25295. 10.1038/s41598-024-76387-w. 39455837 10.1038/s41598-024-76387-w PMC11511857

